# Molecular Characterization and Analysis of 16S Ribosomal DNA in Some Isolates of *Demodex folicullorum*

**Published:** 2017

**Authors:** Afrooz DANESHPARVAR, Gholamreza MOWLAVI, Hamed MIRJALALI, Homa HAJJARAN, Iraj MOBEDI, Saeed Reza NADDAF, Mohammadreza SHIDFAR, Mahsa SADAT MAKKI

**Affiliations:** 1. Dept. of Medical Parasitology and Mycology, School of Public Health, Tehran University of Medical Sciences, Tehran, Iran; 2. Foodborne and Waterborne Diseases Research Center, Research Institute for Gastroenterology and Liver Diseases, Shahid Beheshti University of Medical Sciences, Tehran, Iran; 3. Dept. of Parasitology, Pasteur Institute of Iran, Tehran, Iran; 4. Dept. of Mycology, School of Public Health, Tehran University of Medical Sciences, Tehran, Iran

**Keywords:** Iran, *Demodex folliculorum*, 16S rDNA, Phylogenetic analysis

## Abstract

**Background:**

Demodicosis is one of the most prevalent skin diseases resulting from infestation by *Demodex* mites. This parasite usually inhabits in follicular infundibulum or sebaceous duct and transmits through close contact with an infested host.

**Methods:**

This study was carried from September 2014 to January 2016 at Tehran University of Medical Sciences, Tehran, Iran. DNA extraction and amplification of 16S ribosomal RNA was performed on four isolates, already obtained from four different patients and identified morphologically though clearing with 10% Potassium hydroxide (KOH) and microscopical examination. Amplified fragments from the isolates were compared with GeneBank database and phylogenetic analysis was carried out using MEGA6 software.

**Results:**

A 390 bp fragment of 16S rDNA was obtained in all isolates and analysis of generated sequences showed high similarity with those submitted to GenBank, previously. Intra-species similarity and distance also showed 99.983% and 0.017, respectively, for the studied isolates. Multiple alignments of the isolates showed Single Nucleotide Polymorphisms (SNPs) in 16S rRNA fragment. Phylogenetic analysis revealed that all 4 isolates clustered with other *D. folliculorum,* recovered from GenBank database. Our accession numbers KF875587 and KF875589 showed more similarity together in comparison with two other studied isolates.

**Conclusion:**

Mitochondrial 16S rDNA is one of the most suitable molecular barcodes for identification *D. folliculorum* and this fragment can use for intra-species characterization of the most human-infected mites.

## Introduction

Demodicosis is a term attributed to the skin disorders caused by *Demodex* mites (class Arachnida and order Acarina) (1, 2). The most prevalent species that affect human skin are *D. folliculorum* and *D. brevis.* These species are usually found in follicular infundibulum or sebaceous duct and use sebum as nourishment (3, 4). Newborns can only acquire mites from close contact with infected individuals, but mite survival is low due to low sebum excretion in infants. *Demodex* mite infestation is typically established during the teenage years, and the chance of infestation is correlated with increasing age (5). The reports of infestation by *Demodex* are up to 80% in healthy skin (6, 7) that can increase in elderly people (8). In Iran, studies on Infestation with *Demodex* showing prevalence rate 15.2% in healthy control to 68.3% in patients with clinical manifestations (9–12). Although, different aspects of *Demodex* infestation in human has not been well known yet (13), but the infestation in animals is well established (3, 14). Cutaneous symptoms develop in human gradually due to increasing parasite populations (4, 15). Furthermore, heavy infestations are mostly reported in males compared to females, presumably due to greater number of subcutaneous glands in females (16).

However, species identification and classification is performed based on morphological characterizations in most of laboratories. The most important weaknesses of morphological characterization are lack of sensitivity to identify mix-infestation of simultaneous presence of *D. folliculorum* and *D. brevis* and complexity in characterization of similar but not same mites. On the other hand, molecular techniques can reveal interesting results in subspecies grouping and in the mix-infection (17, 18).

However, although *D. folliculorum* and *D. brevis* are the most prevalent *Demodex* mites reporting from all over the world, but phylogenetic studies have showed that there are several sequence types of both *D. folliculorum* and *D. brevis* (19–22)*.* The current study is the first molecular description of 16S DNA of *Demodex folliculorum* in Iran aimed to characterize and analysis of *Demodex* spp.

## Materials and Methods

### Study population

Samples of *D. folicullorum* included 4 different isolates which had already obtained during September 2014 to January 2016 and were kept out in Dermatology and Leprosy Research Center and Laboratories of Tehran University of Medical Sciences, School of Public Health.

The current study was approved by the Ethic Committee of Tehran University of Medical Sciences. Morphological identification had been performed using Potassium hydroxide 10% solution (KOH) clarification and light microscopy examination (×40 and ×1000 magnifications).

### Molecular analysis

DNA extraction was performed according to the protocol mentioned elsewhere (23) and then achieved DNA purified by Bioneer DNA Extraction Kit. Briefly, 400 μl lysis buffer was added to the mites in microtubes. Each sample was frozen and thawed for four times, then 25 μg/μl Proteinase K was added and all samples were incubated at 60 overnight. Finally, lysate transferred to Bioneer DNA Extraction kit and following steps were conducted according to manufacturer instruction. Extracted DNAs were eluted in Tris-EDTA (pH=7.5) and stored in −20 °C until further use.

### PCR amplification and sequencing

PCR was performed using genus-specific primers, designed, based 16s rRNA gene using online software (http://www.ncbi.nlm.nih.gov/tools/primer-blast/). Following primers 16SF (5′ -GGTATTTTGACTGTGCTAAG 3′) and 16sR (5′- CAATTTTAATAGTCGAACAG 3′) amplified a 390 bp fragment of 16S rDNA. PCR reaction was performed in final volume 25 μl containing 2.5 μl of 10X PCR buffer, 2mM MgCl2, 200μM dNTP, 1.5 unit of Taq polymerase (Fermentase, Thermo Fisher Scientific, Lithuania) and 10 ϱM of each primers. Distillated water and a known positive DNA were used as negative and positive controls, respectively. Amplifications were carried out in PeqLab thermocycler (PEQLAB Biotechnology GmbH, Germany) under conditions: 95 °C for 4 min followed by 35 cycles consisting: 95 °C for 45 sec, 51 °C for 45 sec and 72 °C for 1 min and then a final extension stage at 72 °C for 7 min. PCR products were electrophoresed on 1.5% agarose gel and then were visualized by ethidium bromide staining. Amplicons were purified using the QIAquick Gel Purification Kit (Qiagen, Valencia, CA, USA) and Sequencing was performed by the Sanger method on an ABI 3730 sequencer (Bioneer, Daejeon, South Korea). The resulting sequences were analyzed using BLAST and submitted to GenBank.

### Phylogenetic analysis

Our sequences beside *D. folicullorum, D. canis, D. brevis* and *D. cati* sequences, which were near together in BLAST comparison, were included in molecular analysis. Molecular alignment was performed using by ClustalW in Bioedit software and then phylogenetic tree was constructed using Molecular & Evolution Genetic Analysis software version 6 (MEGA 6) in Maximum-Likelihood test and Tamura 3 parameter model (24). For calculating the reliability of the tree, Bootstrap value with 1000 replication was considered.

## Results

After DNA amplification, a 390 bp fragment of 16S DNA of *D. folicullorum* were obtained in all four isolates. All DNA sequences showed high similarity with other *D. folicullorum*, previously registered in GenBank database. All generated sequences were registered in GenBank database with accession numbers including KF875587, KF875588, KF875589 and KF875590.

**Fig. 1:**
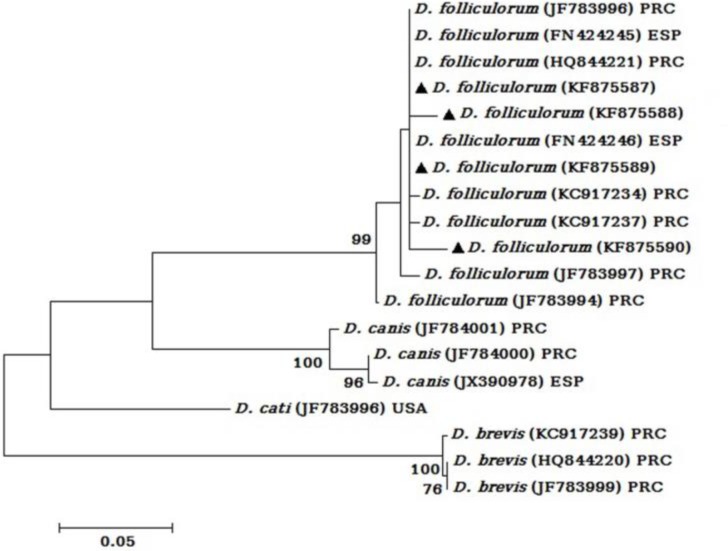
Phylogenetic tree of the mitochondrial 16S ribosomal DNA nucleotide sequences of *D. folliculorum*. The tree was constructed by using the Maximum-Likelihood test and Tamura-3 parameter model in MEGA software version 6. The numbers above the branches indicate the percentage of bootstrap samplings percentages. Branches without numbers have frequencies of less than 75%. Besides the isolates reported in this study, which are marked by a black filled triangle, a number of accession numbers from other studies were applied in phylogenetic analysis. Abbreviations: PRC: People’s Republic of China; USA: United States of America; ESP: Spain

The MEGA 6 software was employed to calculate intra-species distance and similarity. The intra-species distance rate and similarity among the isolates were 0.017 and 99.983%, respectively. Based on phylogenetic analysis, all four isolates placed beside other *D. folliculorum*, recovered from Gene Bank database. In addition, phylogenetic analysis showed that the isolates with accession numbers KF875587 and KF875589 had more similarity together in comparison with two other isolates (Fig. 1).

The tree was constructed by using the Maximum-Likelihood test and Tamura-3 parameter model in MEGA software version 6. The numbers above the branches indicate the percentage of bootstrap samplings percentages. Branches without numbers have frequencies of less than 75%. Besides the isolates reported in this study, which are marked by a black filled triangle, a number of accession numbers from other studies were applied in phylogenetic analysis. Abbreviations: PRC: People’s Republic of China; USA: United States of America; ESP: Spain

**Fig 2:**
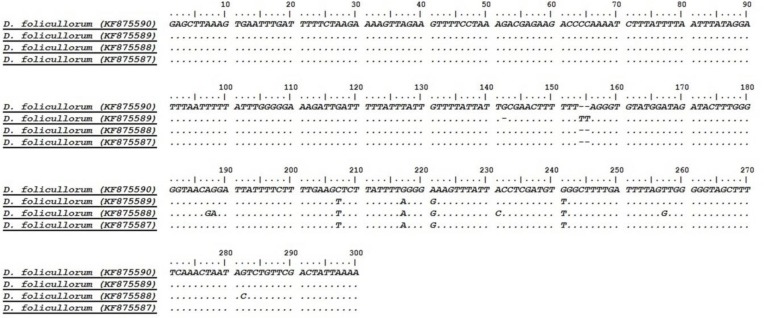
Alignment of mitochondrial 16S ribosomal DNA sequences of *D. folliculorum* isolates. The sequences of this study were aligned by using the ClustalW and Bioedit Software.

Multiple alignment analysis of the isolates was performed and the results showed that the isolate with accession number KF875588 had more Single Nucleotide Polymorphisms (SNPs) throughout the sequenced fragment than other isolates (Fig. 2).

## Discussion

Demodecosis has a wide host range and geographical distribution, all over the world (5, 25). Although classical characterization based on phenotypic properties had been used for years, molecular methods, however, has recently employed to characterize the different species of *Demodex* from the human and animal isolates.

Improving the molecular techniques has provided a new suitable tool for identification of the organism through which ribosomal DNA have considered a characteristic marker for identification and classification of different genera and species of *Demodex* spp., in recent decades (20, 22, 26).

Our study showed that the well-conserved mitochondrial 16S rDNA fragment has some SNPs variation among the same species, already identified as *D. folliculorum* based on morphological properties. In addition, phylogenetic findings revealed that although, all four isolates clustered with other sequences of *D. folliculorum*, retrieved from GenBank database, but there were some SNPs in multi-alignment analysis. This finding is in agreement with other studies that showed, molecular analysis using several mitochondrial genes fragments mitochondrial 16S rDNA could be a useful tool for phylogenetic analysis as well as characterization of not only different species of *Demodex* mites, but also the same species (21, 27–29).

As phylogenetic tree exhibited, in spite of clustering of all *D. folliculorum* isolates together, but there were 8 alleles among *D. folliculorum* of our study as well as those sequences, registered in GenBank database, previously. This criterion of mitochondrial 16S rDNA in *Demodex* mites had also been observed in other studies (26, 30). However, our findings besides all other studies performed on phylogenetic analysis of mitochondrial 16S rDNA showed that this fragment has competence to use as a barcoding tool for distinguishing between *Demodex* species and sub-species analysis.

However, the variations inside this fragment, observed in different studies, are likely attributed to the different colonies that infected hosts, some differences in parasite sites, skin type and source of infection as previously debated (18, 31).

However, the results of sequencing and analysis of the merely ribosomal DNA fragments suggest that other approaches such as Multi Locus Sequencing Test (MLST), which studies several genes’ fragments, are more suitable for clarifying the morphological and genetically feature.

## Conclusion

Mitochondrial 16S rDNA is one of the most suitable molecular barcodes for identification of *D. folliculorum,* also used for iner-species and may be intra-species characterization of the most human-infected mites.
